# Go Micro Aesthetics: A Cross-Sectional Study to Assess Anterior Hard and Soft Tissue Parameters in Young Adults of Bhopal City

**DOI:** 10.7759/cureus.32258

**Published:** 2022-12-06

**Authors:** Bhavika A Bhavsar, Jasleen Suri, Varun Kumar, Vadi Hari Krishna, N Thanigai Selvi, Shantwana Singh

**Affiliations:** 1 Department of Conservative Dentistry and Endodontics, Ram Krishna Dharmarth Foundation (RKDF) Dental College and Research Center, Bhopal, IND

**Keywords:** gingival aesthetic line, zenith point, gingival angle, tooth dimensions, ideal smile, anterior aesthetics

## Abstract

Background

In the maxillary aesthetic zone, the symmetry and proportions of hard and soft dental tissue play a crucial role. A dental practitioner should have a thorough knowledge of parameters such as crown length (CL), crown width (CW), zenith point, and gingival angle (GA) to perform teeth restorations. This study aims to assess the anterior hard and soft tissue parameters and determine the crown shape and its correlation with the gingival parameters of young adults’ anterior teeth.

Methods

In the study, 110 patients were assessed and divided into two groups: Group I with 55 females and Group II with 55 males. Using a calibrated vernier caliper, the following parameters were calculated: crown width (CW)/crown length (CL) ratio, gingival angle (GA), distance from the lateral incisor (LI) to the gingival aesthetic line (GAL), interdental papilla height (PH) between the two central incisors (CI), distance between the central incisor (CI) and the lateral incisor (LI), and distance between LI and canine.

Results

Statistical analysis of data was performed using the independent samples t-test (P<0.05). The mean value of CW, CL, CW/CL ratio, and GA, along with the distance from LIs to GALs and PHs of the anterior teeth, were lower in females than in males. The correlation between the GA and the CW/CL ratio indicated that higher GAs and CW/CL ratios give teeth a square appearance. Moreover, a lower GA and CW/CL ratio will make teeth appear round or narrow.

Conclusion

The present study concluded that the mean values of hard and soft dental tissue parameters are higher in males than in females. These values can be used to restore functional aesthetics in accordance with the results of a local population and could help form restorative, aesthetic, and cosmetic procedures while maintaining focus on the size and shape of teeth and the level of gingiva at various significant treatment stages.

## Introduction

The aim of restorative and aesthetic dentistry is to replicate the natural shape and function of both the clinical crown and periodontal tissue. To achieve good aesthetic results, knowledge about the morphology of the tooth and gingiva is essential. Emphasis should be given to the biological and aesthetic harmony of hard and soft tissue [1]. Aesthetic harmony is determined by various factors, such as symmetry, alignment, shape, size, teeth color, gingival contours, texture, soft tissue height, and emergence profile [2,3].

Clinically, the most apical point of a crown is termed the zenith. It is located 1 mm distal to the midline between the maxillary central incisor and the canine and at the lateral incisor midline [4]. The zenith plays a significant role in diastema treatment, implant insertion, and distal and mesial tooth angulation correction. It is also associated with the gingival angle (GA), which helps determine the shape of teeth and their cervical convergence [2,3]. The size and shape of teeth also differ in accordance with region, gender, race, and diet, which all make an impression on aesthetic appearance. Another aspect that should be considered is the gingiva. The papilla occupies the interdental embrasure between two teeth, and the papilla’s presence or absence can affect a person’s aesthetic appeal [2,5]. Another noticeable feature in aesthetics is the distance of the lateral incisor (LI) from the gingival aesthetic line (GAL).

Aesthetic restorations greatly impact a patient’s smile and appearance, which consequently enhances their self-confidence. The dentogingival complex constitutes an essential component of dentofacial aesthetic restorations [4]. The proportions of a dentofacial complex may look symmetrical from a distance; however, discrepancies may be noticed upon closer inspection. For perfect rehabilitation, these minute discrepancies should be evaluated and corrected.

Various studies have assessed different parameters of the dentofacial region to determine the ideal aesthetics of anterior teeth. Köseoğlu et al. concluded from their study that some smile parameters in the young Turkish population vary according to gender [6]. There were no statistically significant differences observed between males and females, according to the study findings of Melo et al. [7]. With an upward lip curve, an oval tooth shape, and a consonant smile arc and medium smile line, the maxillary interincisal midline was congruent with the facial midline [7]. From their study, Mootha and Jaiswal found that the observed values of width proportions of canine to lateral incisor and lateral incisor to central incisor, and the observed width/height proportion values are in concordance with the values given by Digital Smile Design (DSD®) [8]. A variety of software is available for aesthetic restorations, but it requires specific, advanced skills, and it is not economical. Various methods that require minimal armamentarium and are less technically sensitive also exist. Therefore, it is difficult for operators to choose between a technical or manual approach for the estimation of these parameters. However, according to the literature, both manual and software-based aesthetic designs have resulted in equally aesthetically harmonious and pleasing results, which had a positive impact on the patient and the operator [4]. Therefore, manual methods were used in this research.

In this study, we aimed to determine the correlation between hard and soft dental tissue parameters, which will prove beneficial to anterior aesthetic restoration.

## Materials and methods

Subject selection

For this study, 110 patients were selected, consisting of 55 male and 55 female participants aged between 18 and 30 years. The sample selection was based on the following formula (nt = sample size): *n_t _*= 2 (Z_1-α/2_+Z_1-β_)^2^/*ES.*

Inclusion criteria

The inclusion criteria consist of well-aligned maxillary anterior teeth and healthy gingiva.

Exclusion criteria

The exclusion criteria omitted subjects with the presence of restorations or cavities in the maxillary anterior teeth, a history of periodontal surgery or orthodontic treatment, presence of abrasion, attrition, or erosion, and those taking medication known to increase the risk of gingival hyperplasia. Pregnant or lactating women were also excluded, along with patients with severe medical conditions or compromised immune systems such as patients having human immunodeficiency virus (HIV) or acquired immunodeficiency syndrome (AIDS).

Method

Ethical approval was obtained from the Ethics Committee of Sarvepalli Radhakrishnan University, Bhopal, (reference number: RKDF/DC/PG/2020/14793). A total of 110 patients who visited the Department of Conservative Dentistry and Endodontics for dental treatment were selected as per the inclusion criteria. Informed consent from patients was taken, and the procedure was described to them before the study commenced.

**Figure 1:**
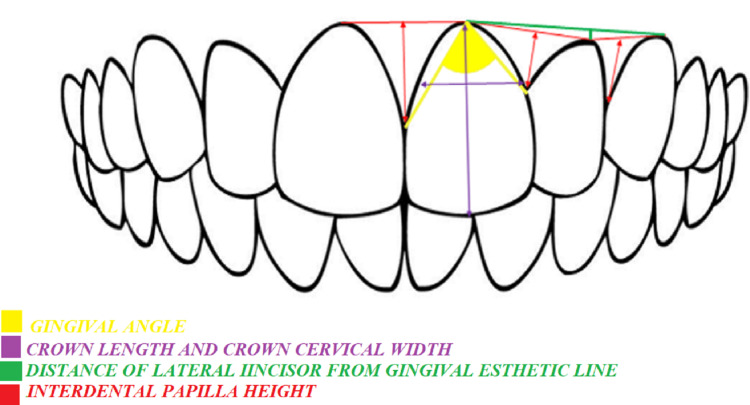
Schematic diagram representing the GA, cervical CW and CL, interdental PH, and distance of the LI from the GAL. GA: gingival angle, CW: crown width, CL: crown length, PH: papilla height, LI: lateral incisor, GAL: gingival aesthetic line

Patients who fulfilled the criteria were then randomly divided into two groups based on their gender: Group I with 55 females and Group II with 55 males. Alginate impressions (Tropicalgin, Zhermack, Italy) were taken that were poured immediately after it was registered, with a type 3 dental stone (Stone Max Dental Stone, Eurosiskin Dental Stone Private Limited, Kottayam, Kerala, India). The cast was trimmed properly, and the landmarks (identified by a trained single investigator) included in the study were marked as depicted in Figure 1.

Assessment of various parameters

Gingival Angle

**Figure 2:**
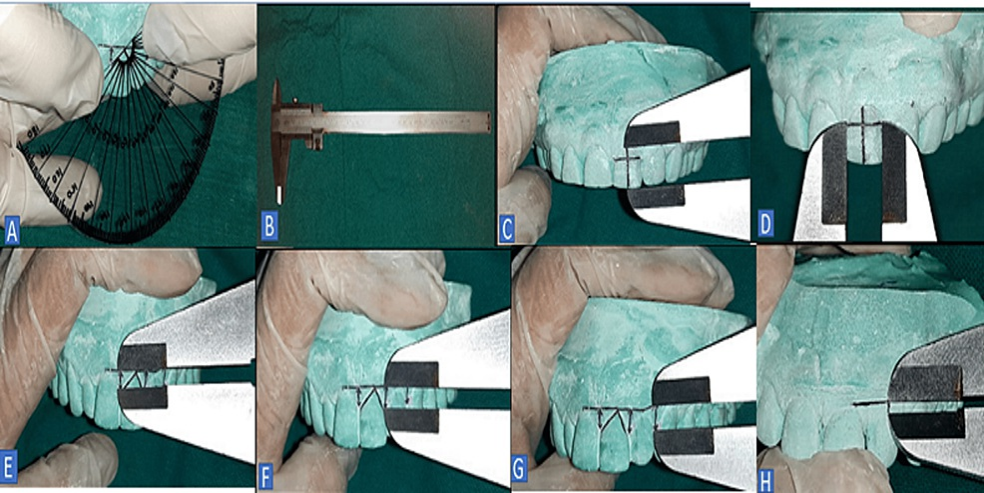
Cast model displaying (A) the GA measured by the customized protractor, (B) the vernier caliper, (C) the measurement of the CL and CW, (E) the PH between the right and the left CIs, (F) the right CI and LI, (G) the right LI and canine, and (H) the distance from the LI to the GAL. GA: gingival angle, CL: crown length, CW: crown width, PH: papilla height, CIs: central incisors, LI: later incisor, GAL: gingival aesthetic line

The gingival angle of the right maxillary central incisor was marked by connecting the zenith point to the mesial and distal contact points (Figure 2A).

Crown Width/Crown Length Ratio

The CW at the incisal edge was calculated. The CW was divided into two halves, and a line was drawn along the long axis of the tooth from the cervical point to the incisal point. This line was measured as the CL (Figure 2C). This CL was evenly divided into three parts (incisal, middle, and cervical). The mesiodistal width of the border between the middle part and the cervical part of the CL was taken as the CW. Then, the CW/CL ratio was calculated (Figure 2D).

Interdental Papilla Height

The zenith points of the right and left CIs, LI, and canine were marked and joined. Then, the PH between two individual teeth was calculated by drawing a line from the GAL to the respective interdental papilla (Figure 2E-2G).

Distance From the Lateral Incisor to the Gingival Aesthetic Line

The GAL was marked by joining the zenith points of the maxillary CI and maxillary canine. A perpendicular line was drawn by joining the zenith point of the LI to the GAL before its distance was measured (Figure 2H).

The maxillary cast used in the present study acts as a three-dimensional structure formed from dental stone. A customized protractor was made on a transparent sheet to measure the exact GA. The highly sensitive photographic method was avoided here, as it would have converted the cast into a two-dimensional structure, which will eventually change the values of all the parameters of the teeth and gingiva (Figure 2B). The other measurements were taken with a vernier caliper (Freemans Vernier Caliper, Freemans, Ludhiana, Punjab, India). A single-blinded study was performed.

Statistical analysis

All measurements were recorded by the same single investigator and statistically evaluated by a statistician. Data were collected from a Microsoft Excel sheet (Microsoft Corp., Redmond, WA, USA), and the results were calculated using an independent samples t-test. With a p-value of <0.05, the results were considered significant. Before the start of the study, a null hypothesis was proposed as the mean of various parameters in both groups (male and female) would be the same.

## Results

Table 1 shows the mean values and standard deviations of all the parameters selected for this study. The mean GA was 89.51º in Group I and 92.16º in Group II, and it was statistically significant (P<0.00001). Moreover, the mean CL was 9.07 mm in Group I and 10.18 mm in Group II. The mean CW was 7.42 mm in Group I and 7.8 mm in Group II. Also, the mean PH between the two maxillary CIs was 4.01 mm in Group I and 4.39 mm in Group II. Furthermore, the mean PH between the LI and the canine was 3.8 mm in Group 1 and 4.38 mm in Group 2. Statistically, these results were highly significant; however, the values for the LI’s distance from the GAL and the interdental papilla length between the CI and the LI were insignificant.

**Table 1:** Intergroup comparison. NS: not significant, S: significant, SD: standard deviation p-value<0.05

Parameters	Group I		Group II		Mean±SD difference	p-value
	Mean	SD	Mean	SD		
Gingival angle (degree)	89.51	1.26	92.16	2.75	2.60±1.44	<0.000 (S)
Crown length (mm)	9.07	1.02	10.18	1.59	1.06±0.51	0.000 (S)
Crown width (mm)	7.42	0.6	7.8	0.83	0.40±0.28	0.003 (S)
Lateral incisor distance from the gingival aesthetic line	0.79	0.49	1.02	0.94	0.30±0.43	0.056 (NS)
Interdental papilla length between two maxillary central incisors	4.01	0.47	4.39	0.47	0.34±0.04	0.000 (S)
Interdental papilla length between the central and the lateral incisor	3.54	0.74	3.64	0.57	0.15±0.21	0.195 (NS)
Interdental papilla length between the lateral incisor and the canine	3.8	0.52	4.38	0.68	0.55±0.13	<0.000 (S)

Table 2 indicates an intragroup comparison determining the correlation between the GA and the CW/CL ratio to establish the shape of teeth.

**Table 2:** Intragroup comparison of Group I and Group II. S: significant, SD: standard deviation p-value<0.05

Tooth shape		CW/CL ratio (mean±SD)		Gingival angle (mean±SD)	
Group I	Group II	Group I	Group II	Group I	Group II
Tapered (58.15%)	Tapered (27.27%)	0.56±0.81	0.60±1.12	88.28±2.16	91.33±3.12
Ovoid (32.72%)	Ovoid (63.63%)	0.33±1.96	0.78±2.61	89.6±1.21	91.66±3.24
Square (10%)	Square (10%)	0.094±1.29	1.03±2.01	90.1±2.39	94.23±4.37
p-value	p-value	<0.000 (S)	<0.000 (S)	<0.000 (S)	<0.000 (S)

**Figure 3:**
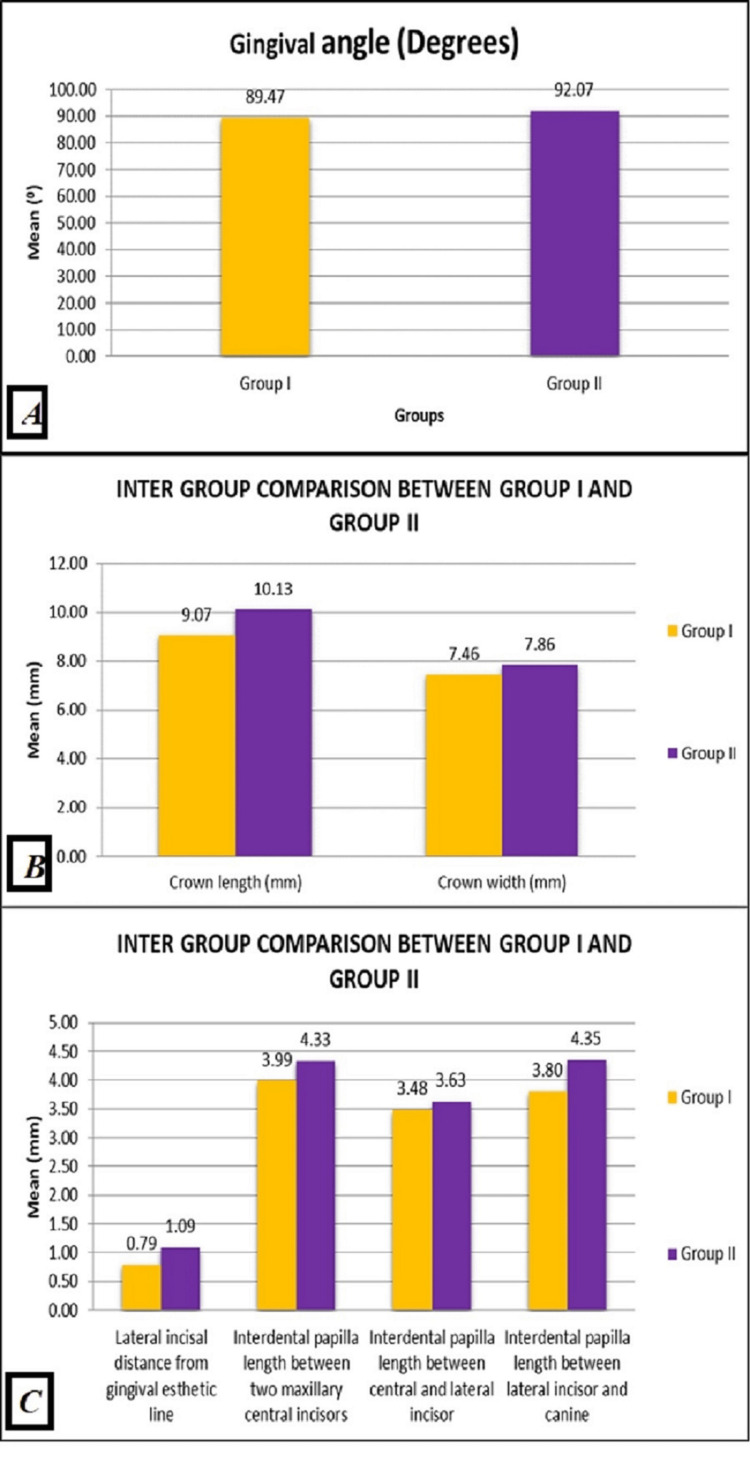
(A) The mean value of GA variation in Group I and Group II. (B) The mean value of CL and CW variation in Group I and Group II. (C) The mean value of the distances from the LI to the GAL and PH between Group I and Group II. GA: gingival angle, CL: crown length, CW: crown width, LI: later incisor, GAL: gingival aesthetic line, PH: papilla height

With high clinical significance, the mean GA (Figure 3A), CL, and CW (Figure 3B) were all found in males more than in females (Figure 3A). The mean of the LI from the GAL was also discovered in males more than in females but with no relevant significance. Moreover, the mean PHs between the right and left CI, right CI and LI, and LI and canine were found more in males than in females; however, the PHs between the right and left CI and the LI and the canine were only clinically significant (Figure 3C).

Regarding the correlation between the GA and the CW/CL ratio in the two groups, as the CW/CL ratio increased, there was a high significance in the GA. Tapered teeth have a low CW/CL ratio and low GA, whereas square teeth have a high CW/CL ratio and high GA.

## Discussion

In recent times, patients demand an ideal aesthetic appearance of teeth. To achieve an ideal aesthetic dental appearance, the pressure among dentists has increased to provide ideal results. Knowledge of gingival tissues and tooth relation lays the foundation for aesthetic and restorative dentistry. Therefore, this study was conducted to analyze the dimensions of clinical crown and soft tissues, which could help determine the crown shape and its correlation with gingival parameters of young adults’ anterior teeth.

Various methods can be carried out to analyze hard and soft dental tissues. Both manual and digital methods have been shown to give accurate tooth discrepancy results. In this study, manual methods (i.e., a vernier caliper and a customized protractor) were used [9], since the measurements of the cast can be done in a three-dimensional form. Correi et al. concluded from their study that both manual and digital three-dimensional scanning methods are reliable for assessing tooth size discrepancy [10].

The proportions of gingival tissues can make a significant difference to the appearance and aesthetics of maxillary anterior teeth. An asymmetric appearance will not only give an unappealing look but also disrupt tooth function. The scalloping of gingival tissue at the cement-enamel junction provides the zenith point, which determines the inclination and emerging profile of a tooth [1]. The present study shows that a higher GA is more common in males than in females. Also, it affirms that, in both males and females, the higher the GA, the squarer the teeth, and the lower the GA, the triangular or oval the teeth. In a similar study, Kolte et al. concluded that the GA in females was smaller than in males [1]. Furthermore, Gobbato et al. stated that the higher the GA, the broader or squarer the teeth and the lower the GA, the more triangular the teeth [3]. Olsson et al. studied the relationship between tooth dimension and gingival biotype and stated that long, narrow teeth will have a thinner gingival biotype [11], which will lessen the GA.

This emergence of tooth shape plays a dominant role in anterior aesthetics [5] and is related to the CW and CL of anterior teeth [2,3]. The greater the CW/CL ratio, the squarer the teeth, and the smaller the CW/CL ratio, the narrower the teeth. Similar to our study, Sridhar et al.’s research on the Chennai population showed that the cervico-incisal height and mesiodistal width are generally larger for male teeth than for female teeth [12]. Singh and Goyal concluded that the mesiodistal crown dimensions of male teeth are larger than those of females [13]. Abraham and Athira stated that long, slender teeth are associated with thin gingival biotypes and are more prone to recession [14].

Tarnow et al. discovered that when the distance between the level of the crestal bone and the most apical contact point of the tooth was less than 5 mm, the interproximal space was filled by interdental papilla. If the interdental papilla does not fill the interproximal space, there is a black triangular appearance. In such conditions, to produce a symmetrical appearance of teeth and gingiva, the restorative and reconstructive approach should be designed to fill the black triangular space through the desired treatment (e.g., pink composite and surgical intervention) [15]. Murthy and Ramani, in their study, have shown that the golden proportion (i.e., the correct proportion of the anterior teeth visible for an ideal smile) is only found in 14%-25% of people [16].

These soft tissue analyses can help a dental practitioner form an accurate treatment plan. Available treatment options for appropriate cases include tooth-colored composites [13], gingival veneers [14], and gingivectomies.

Combining the understanding of the morphological structure of the teeth’s hard and soft tissues in relation to the anterior anatomic region, the materials used to repair, reconstruct, or replace a tooth or gingival structure, and the artistic skills of the dental practitioner will play a major role in a practitioner’s capability to meet the challenges of specific patient requirements and provide the desired result of aesthetically appealing anterior teeth.

The current study found that the dimension of male teeth was larger than that of female teeth. This leads to the conclusion that males have wider teeth than females, and thus, the null hypothesis was rejected.

The present study had some limitations. It was conducted on dental casts, not on patients directly, so there could be a variation in the exact dimensions of the various calculated parameters, the present study was restricted to the population of Bhopal only, and the calculation cannot be justified in patients requiring different treatment options (e.g., composite restorations, fixed prosthesis, or any variations in the treatment).

Further studies with larger samples are needed to validate the results. In our study, we used manual methods for measuring the various parameters; however, more specific or accurate methods, such as digital processes, could have been used for this task.

## Conclusions

The current study showed that the dimension of the male teeth was larger than that of the female teeth. Aesthetic parameters can be assessed either manually or by using software. The soft and hard tissue measurements could help dentists attain the required degree, width, and length of anatomic parameters to provide the desired aesthetic results. Crown length, crown width, zenith points, and other soft and hard tissue parameters help in aesthetic smile design.
